# Virus-Induced Tubules: A Vehicle for Spread of Virions into Ovary Oocyte Cells of an Insect Vector

**DOI:** 10.3389/fmicb.2017.00475

**Published:** 2017-03-22

**Authors:** Zhenfeng Liao, Qianzhuo Mao, Jiajia Li, Chengcong Lu, Wei Wu, Hongyan Chen, Qian Chen, Dongsheng Jia, Taiyun Wei

**Affiliations:** State Key Laboratory of Ecological Pest Control for Fujian and Taiwan Crops, Institute of Plant Virology, Fujian Agriculture and Forestry UniversityFuzhou, China

**Keywords:** plant reoviruses, rice gall dwarf virus, Pns11 tubules, insect vector, transovarial transmission, ovary microvilli, follicular cells, oocyte

## Abstract

Many arthropod-borne viruses are persistently propagated and transovarially transmitted by female insect vectors through eggs, but the mechanism remains poorly understood. Insect oocytes are surrounded by a layer of follicular cells, which are connected to the oocyte through actin-based microvilli. Here, we demonstrate that a plant reovirus, rice gall dwarf virus (RGDV), exploits virus-containing tubules composed of viral non-structural protein Pns11 to pass through actin-based junctions between follicular cells or through actin-based microvilli from follicular cells into oocyte of its leafhopper vector *Recilia dorsalis*, thus overcoming transovarial transmission barriers. We further determine that the association of Pns11 tubules with actin-based cellular junctions or microvilli of the ovary is mediated by a specific interaction between Pns11 and actin. Interestingly, RGDV can replicate and assemble progeny virions in the oocyte cytoplasm. The destruction of the tubule assembly by RNA interference with synthesized double-stranded RNA targeting the Pns11 gene strongly inhibits transovarial transmission of RGDV by its vectors. For the first time, we show that a virus can exploit virus-induced tubule as a vehicle to overcome the transovarial transmission barrier by insect vectors.

## Introduction

Many arthropod-borne animal viruses (arboviruses) or plant viruses that cause significant global health as well as agricultural problems are vertically transmitted by female insects through eggs in a process known as transovarial transmission (Hogenhout et al., 2008; Huo et al., 2014). Rice dwarf virus (RDV), a plant reovirus, is the first plant virus recorded to be transmitted transovarially by insect vectors (Fukushi, 1933). Generally, transovarial transmission can facilitate the persistence of viral pathogens in nature, especially during periods unfavorable for horizontal transmission (Huo et al., 2014; Lequime and Lambrechts, 2014). However, the mechanisms for transovarial transmission of viruses by insect vectors remain poorly understood.

The insect ovary is composed of ovarioles, and each ovariole contains a germarium, vitellarium, and pedicel from apex to base (Szklarzewicz et al., 2007). The oocytes produced by the germarium are arranged linearly in the vitellarium and surrounded by a layer of follicular cells (Szklarzewicz et al., 2007). The plasma membrane of the follicular cell facing the oocyte has abundant actin-based microvilli (Swiatek, 2006). Current data suggest that viral pathogens themselves do not have the ability to enter the oocyte, but instead use the existing oocyte entry pathways in insects (Huo et al., 2014). For example, rice stripe virus (RSV), a tenuivirus, enters the oocyte along with vitellogenin (Vg), a protein synthesized by the fat body and transported into the growing oocytes via receptor-mediated endocytosis (Huo et al., 2014). However, whether this is a common mechanism for viruses to enter the oocyte or whether viruses develop different strategies, is unknown.

Transovarial transmission is often associated with the replication of propagative viruses in insect vectors (Hogenhout et al., 2008). Viral inclusions induced by the replication of these persistent viruses can facilitate viral spread in their insect vectors (Hogenhout et al., 2008). For example, plant reoviruses can exploit virus-containing tubules constructed by viral non-structural proteins to either pass through the actin-based microvilli of the midgut epithelium into the lumen, or across the basal lamina from the midgut epithelium into the visceral circular muscle of leafhopper or planthopper vectors (Chen et al., 2012; Jia et al., 2014). Actin filaments are recruited to enclose the tubules through the direct interaction of these viral non-structural proteins with actin and actin-binding proteins (Mar et al., 2014; Wei and Li, 2016). The actin filaments form a “lighted rocket” to propel tubules to overcome various membrane or tissue barriers in insect vectors (Wei and Li, 2016). Such a viral spread mechanism has been termed actin-based tubule motility (ABTM) (Wei and Li, 2016). We predict that ABTM may be used by plant reoviruses to overcome the transovarial transmission barriers of insect vectors.

Here, we used a plant reovirus, rice gall dwarf virus (RGDV), and its main insect vector, leafhopper *Recilia dorsalis*, to study how a propagative virus overcomes the transovarial transmission barrier by utilizing virus-induced tubules. RGDV, which causes substantial yield loss in southern China and Southeast Asia, was first described in 1979 in Thailand (Omura et al., 1980; Zheng et al., 2015). RGDV is transmitted by leafhopper vectors in a persistent-propagative and transovarial manner (Omura et al., 1980; Inoue and Omura, 1982). Its virion is an icosahedral particle approximately 65–70 nm in diameter with two concentric layers of proteins that enclose the core (Miyazaki et al., 2005). The viral genome consists of 12 segments of dsRNAs that encode six structural and six non-structural proteins (Moriyasu et al., 2007; Zhang et al., 2008). The non-structural protein Pns9 can form a viroplasm-matrix for viral replication and assembly of progeny virions in insect vectors (Akita et al., 2011; Zheng et al., 2015). Viral non-structural protein Pns11, the primary component of virus-containing tubules (Pns11 tubules), can facilitate intercellular spread of RGDV among cultured insect vector cells (Wei et al., 2009; Chen et al., 2013). However, its role in viral transovarial transmission is unknown. In this study, we find that about a quarter of viruliferous *R. dorsalis* females can transovarially transmit RGDV to their offspring. Furthermore, RGDV enters the ovary oocytes of *R. dorsalis* using ABTM to achieve transovarial transmission.

## Materials and Methods

### Insects, Virus, Cells, and Antibodies

Leafhoppers *R. dorsalis* were collected from Guangdong Province in southern China and reared on rice plants. The RGDV samples were collected from rice fields from Guandong Province in southern China. Continuous monolayer cultures of vector cells (VCMs) were developed from *R. dorsalis* and maintained on growth medium as described previously (Chen et al., 2013). Rabbit polyclonal antibodies against intact viral particles, major outer capsid protein P8, and non-structural proteins Pns9 and Pns11 of RDGV were prepared as described previously (Wei et al., 2009; Chen et al., 2013). IgGs, isolated from the polyclonal antibodies, were conjugated to fluorescein isothiocyanate (FITC) or rhodamine (Invitrogen) according to the manufacturer’s instructions.

### Double Labeling of Pns11 and Actin in VCMs during Infection by RGDV

VCMs derived from *R. dorsalis* were synchronously inoculated by RGDV as described previously (Chen et al., 2013). At 84 h post inoculation (hpi), VCMs were fixed, immunolabeled with Pns11-specific IgG conjugated to FITC (Pns11-FITC) and the actin dye phalloidin-rhodamine (Invitrogen), and then processed for immunofluorescence microscopy as already described (Chen et al., 2012). As controls, mock-infected VCMs were treated in the same way.

### Immunofluorescence Labeling of Leafhopper Ovaries after Viral Infection

Five hundred second-instar nymphs of *R. dorsalis* were fed on RGDV-infected rice plants for 2 days, then transferred to healthy rice plants. On different days after adult eclosion, the female ovaries or intestines were dissected, fixed, immunolabeled with viral particles-specific IgG conjugated to rhodamine (virus-rhodamine) and actin dye phalloidin-FITC, or with Pns11-specific IgG conjugated to rhodamine (Pns11-rhodamine) and actin dye phalloidin-FITC, and processed for immunofluorescence microscopy, as described previously (Zheng et al., 2015). As a control, non-viruliferous ovaries were dissected from leafhoppers that had fed on healthy rice plants and treated in the same way.

### Electron Microscopy

For electron microscopy, the ovaries dissected from viruliferous insects were fixed, dehydrated and embedded as described previously (Wei et al., 2006). For immunogold labeling, the ultrathin sections were incubated with viral particles-specific or Pns9-specific IgGs and immunogold-labeled goat antibodies against rabbit IgG that had been conjugated with 15-nm gold particles (ABcam), as described previously (Wei et al., 2006). Sections were imaged with a transmission electron microscope (H-7650, HITACHI).

### Yeast Two-Hybrid Assay

A yeast two-hybrid assay was performed using a DUALmembrane starter kit (DUALsystems Biotech) according to the manufacturer’s instructions. Pns11 gene of RGDV (GenBank accession number: AB030009.1) was cloned into the bait vector PBT-STE, and the actin gene of *R. dorsalis* (GenBank accession number: AMD16549.1) was cloned into the prey vector pPR3-N. The recombinant plasmids PBT-STE-Pns11 and pPR3-N-actin, and the bait construct pPR3-N were used to transform *Saccharomyces cerevisiae* (strain NMY51). Plasmids NubG-Fe65 (positive control) and pPR3-N (negative control) were used to transform NMY51 as controls. All transformants were grown on synthetic dropout (SD)-Trp-Leu-His-Ade agar plates for 3–4 days at 30°C.

### Pull-Down Assay

The Pns11 and GFP genes were amplified and cloned into a pDEST17 vector, which included a His-tag. A GST-fused actin construct was generated by cloning the *R. dorsalis* actin mRNA sequence into the pGEX-3X vector. All recombinant proteins were expressed in *Escherichia coli* strain BL21. Lysates of *E. coli* expressing Actin-GST was incubated with glutathione-Sepharose beads at 4°C for 4 h. Lysates of *E. coli* expressing His-Pns11 or His-GFP were added to the beads and incubated for 4 h at 4°C. The mixture was collected and washed with wash buffer at 4°C. Immunoprecipitated proteins were separated by SDS-PAGE and detected by a Western blot assay with His-tagged and GST-tagged antibodies (Sigma), respectively.

### Knockdown of Pns11 Gene Expression by RNA Interference (RNAi) Induced by Microinjection of Synthesized dsRNAs into Viruliferous *R. dorsalis*

The dsRNAs targeting the Pns11 gene (dsPns11) or GFP gene (dsGFP) were synthesized as previously reported (Chen et al., 2013). Second-instar nymphs of *R. dorsalis* were fed on RGDV-infected rice plants for 2 days, then transferred to healthy rice plants. Newly emerged *R. dorsalis* adults were microinjected with 0.5 nL dsRNAs (0.5 μg/μl). To collect viruliferous insects, at 4 days after microinjection, the female intestines were dissected, fixed, immunolabeled with virus-rhodamine, as described previously (Zheng et al., 2015). The ovaries were then dissected from the females with virus-infected intestines. The total RNAs of ovaries were extracted using Trizol reagent (Invitrogen, USA), and then the transcript levels of Pns11 and P8 genes of RGDV were quantified by relative RT-qPCR assay with the SYBR Green PCR MasterMix kit (Promega, USA) in a Mastercycler Realplex4 real-time PCR system (Eppendorf, German), as described previously (Lan et al., 2016). The relative abundance of the Pns11 and P8 genes was normalized to an internal control gene actin and estimated by the 2^-∆∆Ct^ (cycle threshold) method. The significance of comparisons among the relative expression levels of these two genes was analyzed with a one-way analysis of variance with Tukey’s multiple comparison using SPSS 13.0 software (SPSS Inc., Chicago, IL, USA). The sequences of primers were available in the Supplementary Table 1.

The accumulation of Pns11 or P8 of RGDV in the ovaries was also analyzed via Western blotting with antibodies against Pns11 or P8, respectively. The dissected ovaries were also immunolabeled with viral particles-specific IgG conjugated to FITC (virus-FITC) and Pns11-rhodamine, and then processed for immunofluorescence microscopy, as described previously (Chen et al., 2013). To determine whether dsPns11 treatment inhibited transovarial transmission, the dsPns11- or dsGFP-treated virgin females were crossed to healthy males in a glass test tube containing rice seeding. After laying the eggs, the viruliferous females were selected by RT-PCR assay using RGDV P8 primers. The offspring of each cross from viruliferous female and healthy male were tested for RGDV by RT-PCR assay.

## Results

### Sequential Infection of RGDV in the Ovary of *R. dorsalis*

**FIGURE 1:**
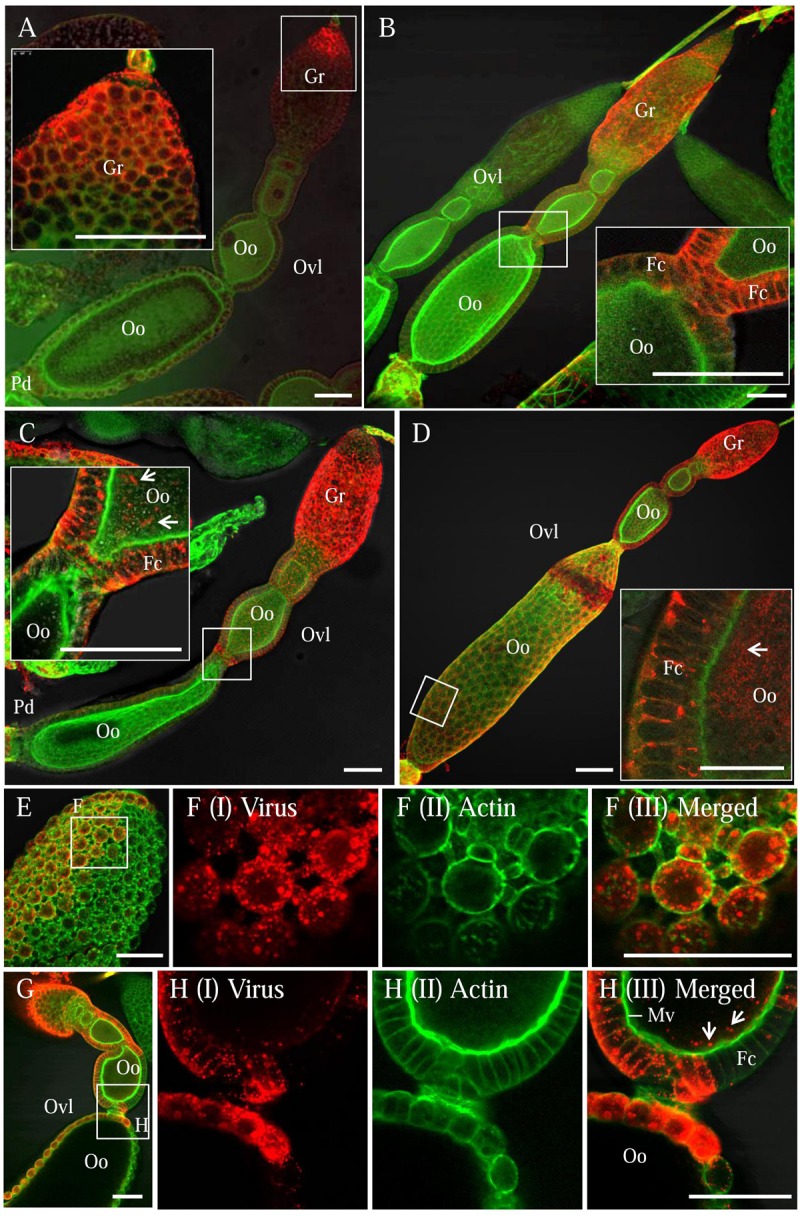
**Infection route of RGDV in the ovary of viruliferous *R. dorsalis.*** Viruliferous ovaries were immunolabeled with actin dye phalloidin-FITC (green) and virus-rhodamine (red) and examined with immunofluorescence microscopy. **(A)** At 3 days after eclosion, RGDV initially invaded the germ cells of germarium at the anterior end of ovariole. **(B)** At 5 days after eclosion, RGDV was present throughout the germarium and had spread toward the follicular cells surrounding the oocyte. **(C)** At 7 days after eclosion, RGDV was present in the cytoplasm of the developing oocyte. **(D)** By 9 days after eclosion, the whole ovariole was infected, and RGDV had invaded the mature oocyte near the pedicel. Insets in **(A–D)** were enlargements of the boxed areas. **(E,F)** Viral accumulation and spread among the follicular cells. **(F)** An enlargements of the boxed area in **(E)** to show the spread of RGDV across the junctions between follicular cells. **(G,H)** RGDV spread from the follicular cells to the developing oocytes by passing through the microvilli. **(H)** Enlarged images of the boxed area in **(G)**. White arrows showed viral particles in the developing oocyte. Fc, follicular cell; Gr, germarium; Oo, oocyte; Pd, pedicel; Ovl, ovariole. Bars: 70 μm. All immunofluorescence figures are representative of at least three repetitions.

We first dissected the ovaries of *R. dorsalis* at different days after eclosion and immunolabeled them with actin dye phalloidin-FITC and virus-rhodamine to follow RGDV infection. At 3 days after eclosion, RGDV was first present in the germarium at the anterior end of the ovariole in 11% of leafhoppers (**Figure 1A** and **Table 1**). At 5 days after eclosion, viruses had spread toward the pedicel among the follicular cells in about 10% of leafhoppers (**Figure 1B** and **Table 1**). At 7 days after eclosion, we observed that RGDV had spread into the developing oocyte in about 12% of leafhoppers (**Figure 1C** and **Table 1**). At 9 days after eclosion, about 21% leafhopper ovaries were infected by RGDV, but viruses had entered the mature oocyte near the pedicel in only about one-third of virus-infected ovaries (**Figure 1D** and **Table 1**). At this time, viruses were present in the intestines in about 80% of leafhoppers tested (**Table 1**). Careful observation revealed that RGDV spread across the junctions between follicular cells (**Figures 1E,F**). Meanwhile, RGDV can spread from the follicular cells to the oocytes by passing through the microvilli (**Figures 1G,H**). Taken together, these results indicated that RGDV first entered the germarium, proceeded to the follicular cells, and finally into the oocytes of its female vectors. Furthermore, RGDV may encounter strong barriers in its path from the germarium to the mature oocyte.

**Table 1:** Occurrence of RGDV antigens in leafhopper intestines or ovaries as detected by immunofluorescence microscopy.

Tissues examined	No. of insects positive for viral antigen in intestines or ovaries at different days after eclosion (*n* = 100)			
	3 days	5 days	7 days	9 days
Germarium	11	14	17	21
Follicular cell	0	10	16	21
Developing oocyte	0	0	12	14
Mature oocyte	0	0	0	7
Intestine	50	56	72	80


### Pns11 Tubules Facilitated Viral Spread into the Oocytes of *R. dorsalis*

**FIGURE 2:**
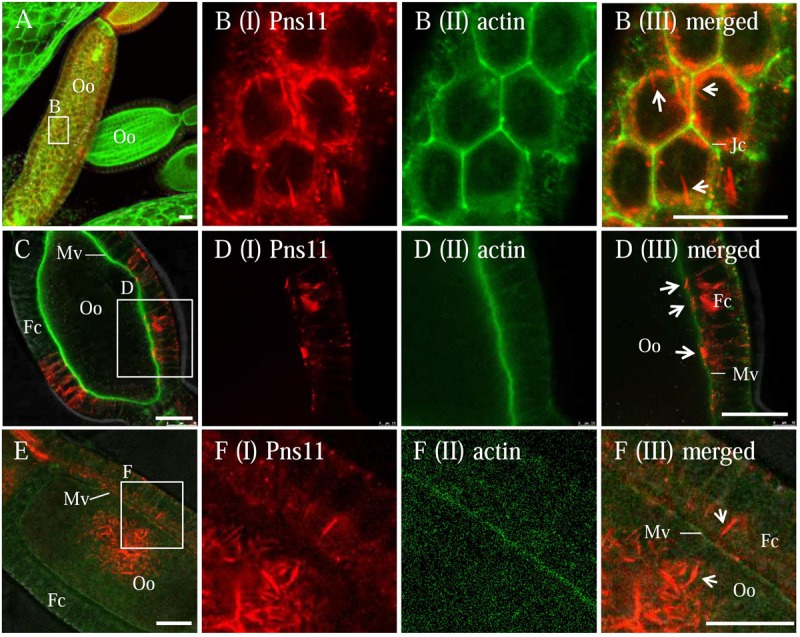
**Distribution of Pns11 tubules in the ovary of viruliferous *R. dorsalis*.** Viruliferous ovaries were immunolabeled with actin dye phalloidin-FITC (green) and Pns11-rhodamine (red) and examined with immunofluorescence microscopy. **(A,B)** Spread of Pns11 tubules (white arrows) across actin-based junctions of follicular cells. **(B)** Enlargements of the boxed area in **(A)**. **(C–F)** The association of Pns11 tubules (white arrows) with actin-based microvilli between follicular cells and oocyte. **(D,F)** Enlargements of the boxed areas in **(C)** and **(F)**, respectively. Fc, follicular cell; Jc, junctions; Mv, microvilli; Oo, oocyte; Ovl, ovariole. Bars: 30 μm. All immunofluorescence figures are representative of at least three repetitions.

Our previous study found that RGDV can exploit virus-containing Pns11 tubules to spread among cultured insect vector cells by passing through the actin-based cellular protrusions (Chen et al., 2013). To test whether RGDV can exploit Pns11 tubules to move among follicular cells or from follicular cells to developing oocytes, immunofluorescence microscopy was used to observe the distribution of Pns11 tubules in virus-infected ovaries by immunolabeling with Pns11-rhodamine and actin dye phalloidin-FITC. We observed the accumulation of Pns11 tubules in the follicular cells (**Figures 2A–C**). Such Pns11 tubules were observed to closely associate with the actin-based junctions between adjacent follicular cells (**Figure 2B**), or with the actin-based microvilli between follicular cells and oocyte (**Figure 2D**). Finally, abundant Pns11 tubules accumulated in the oocyte (**Figures 2E,F**). Seemingly, Pns11 tubules can mediate viral spread from follicular cells into oocyte by passing through actin-based microvilli.

**FIGURE 3:**
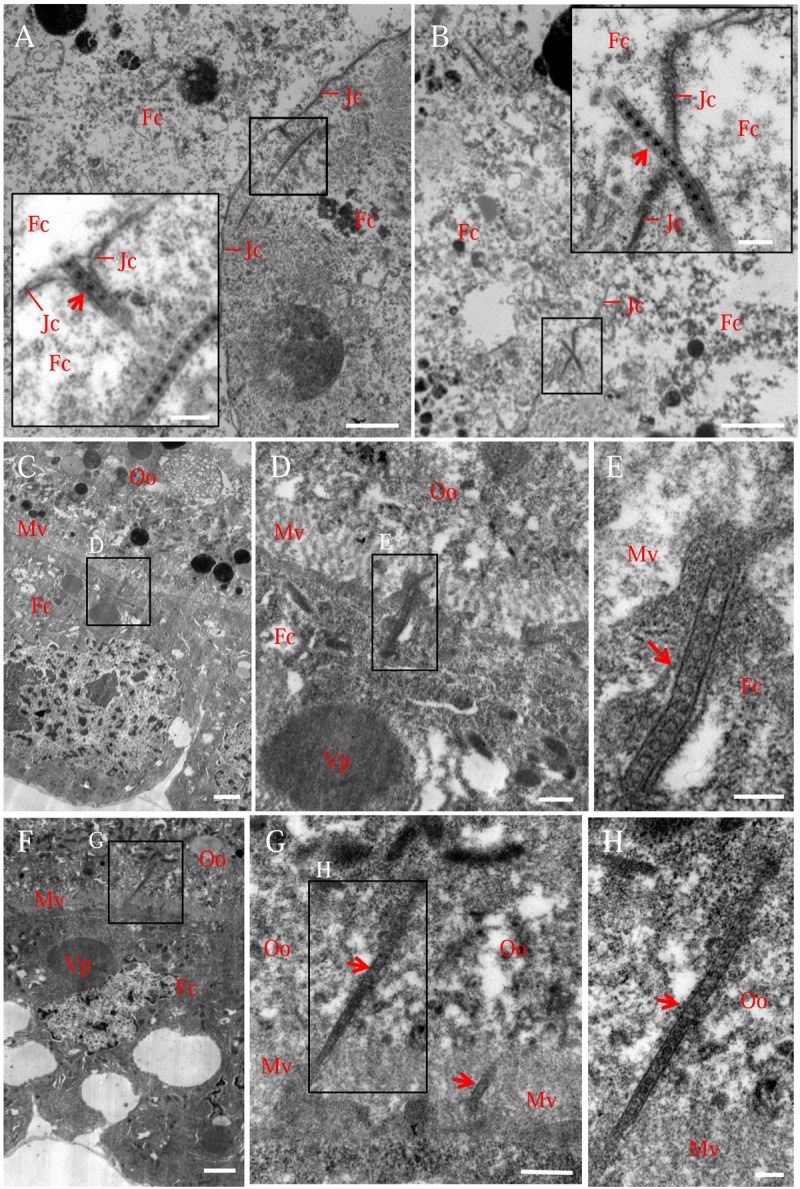
**Electron micrographs showing virus-containing tubules in the vector ovary. (A,B)** Virus-containing tubules (arrows) passed through the junctions of follicular cells. Insets in **(A,B)** were enlargements of the boxed areas. **(C–E)** Virus-containing tubules (arrows) protruded from the cytoplasm of follicular cell toward developing oocyte. **(F–H)** Virus-containing tubules (arrows) inserted into the microvilli of oocyte. **(D,G)** Enlargements of the boxed areas in **(C,F)**, respectively. **(E,H)** Enlargements of the boxed areas in **(D,G)**, respectively. Fc, follicular cell; Jc, junction complex; Mv, microvilli; Oo, oocyte; Vp, viroplasm. Bars in **(A–C,F)**: 2 μm; Bars in insets of **(A,B)**: 200 nm; Bars in **(D,G)**: 500 nm; Bars in **(E,H)**: 200 nm. All electron micrographs are representative of at least three repetitions.

Electron microscopy confirmed that the association of virus-containing tubules with the junctions of follicular cells or with the microvilli of oocyte (**Figure 3**). We observed that one end of the tubule made contact with the side of the junction, then the extension of the tubule finally penetrated the junction, leading to the spread of virions from one follicular cell to adjacent one (**Figures 3A,B**). We also observed that the tubule extended into the plasma membrane of follicular cells toward the oocyte (**Figures 3C–E**), then crossed the microvilli into the oocyte cytoplasm (**Figures 3F–H**), leading to the spread of virions into the oocyte. Free viral particles were not found to associate with the junctions or microvilli of the insect ovaries (**Figure 3**). Taken together, our results suggested that Pns11 tubules can guide and escort viral particles to spread among follicular cells and into the oocyte.

**FIGURE 4:**
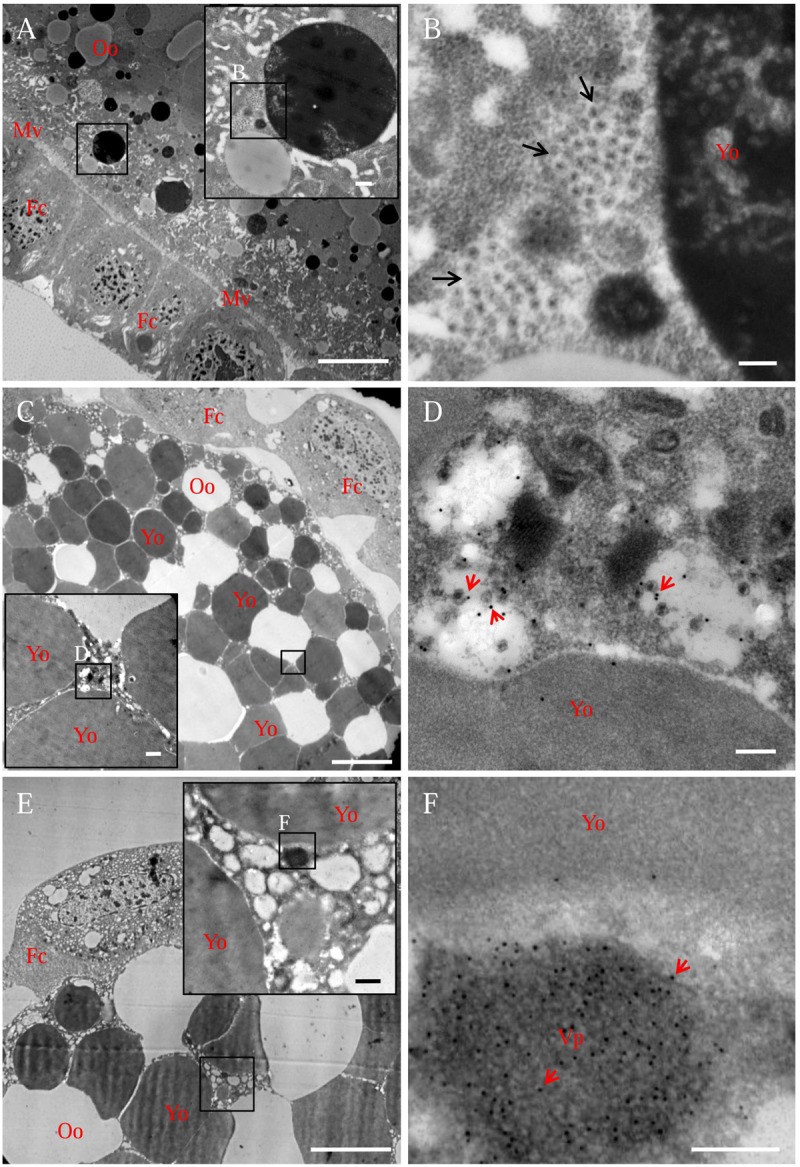
**Electron micrographs showing the subcellular localization of viral particles and non-structural protein Pns9 of RGDV in oocyte cytoplasm of viruliferous *R. dorsalis*. (A,B)** Distribution of viral particles in the oocyte cytoplasm. Inset in **(A)** was an enlargement of the boxed area. **(B)** An enlargement of the boxed area in the inset of **(A)**, showing viral particles (arrows) accumulated at the periphery of yolk granules. **(C,D)** Viral particles in the oocyte cytoplasm. Ovaries were immunolabeled with viral-particle-specific IgG as primary antibody, followed by treatment with 15-nm gold-particle-conjugated goat antibodies against rabbit IgG as secondary antibody. Inset in **(C)** was an enlargement of the boxed area. **(D)** An enlargement of the boxed area in the inset of **(C)**, showing gold particles (arrows) on viral particles. **(E,F)** Pns9-specific viroplasm in the oocyte cytoplasm. Ovaries were immunolabeled with Pns9-specific IgG as primary antibody, followed by treatment with 15-nm gold-particle-conjugated goat antibodies against rabbit IgG as secondary antibody. Inset in **(E)** was an enlargement of the boxed area. **(F)** An enlargement of the boxed area in the inset of **(E)**, showing gold particles (arrows) on viroplasm matrix. Fc, follicular cell; Mv, microvilli; Oo, oocyte; Vp, viroplasm; Yo, yolk granule. Bars in **(A,C,E)**: 10 μm; Bars in insets of **(A,C,E)**: 500 nm; Bars in **(B,D,F)**: 200 nm. All electron micrographs are representative of at least three repetitions.

In the developing oocyte cytoplasm, viral particles usually accumulated at the periphery of the yolk granule (**Figures 4A,B**). Immunoelectron microscopy confirmed that viral particles-specific IgG recognized these viral particles (**Figures 4C,D**). Furthermore, viral non-structural protein Pns9-specifc IgG specifically recognized viral inclusions, namely, the viroplasm, in the oocyte cytoplasm (**Figures 4E,F**). Because the viroplasm was the site for viral replication and assembly of progeny virions (Wei et al., 2009; Akita et al., 2011), we thus determined that RGDV can propagate in the oocyte cytoplasm.

### RGDV Pns11 Directly Interacted with Actin of *R. dorsalis*

**FIGURE 5:**
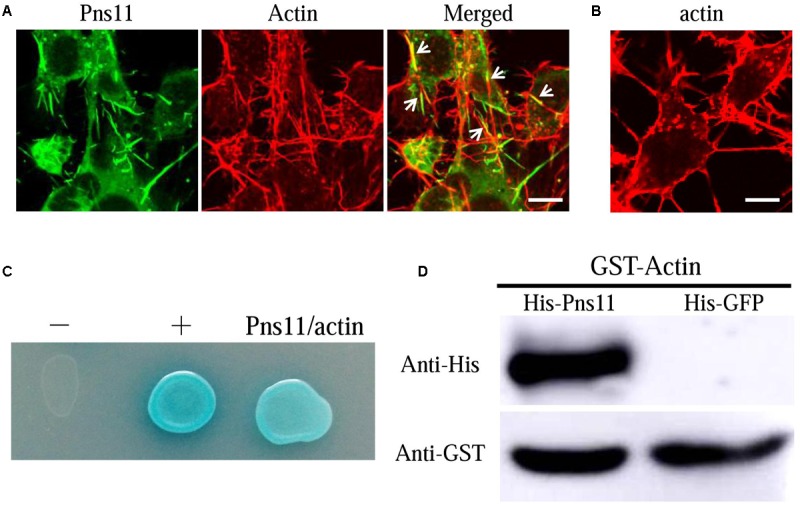
**Association of Pns11 tubules with actin of *R. dorsalis*. (A,B)** Immunofluorescence micrographs of the colocalization of Pns11 tubules with actin filaments (arrows) in VCMs at 48 hpi. RGDV-infected **(A)** and mock-infected **(B)** VCMs were immunolabeled with actin dye phalloidin-rhodamine (red) and Pns11-FITC (green). Bars: 5 μm. **(C)** Interaction between Pns11 of RGDV and actin of *R. dorsalis*, as detected by yeast two-hybrid assay. Transformants on an SD-Trp-Leu-His-Ade agar plate were shown. –, negative control, i.e., pBT3-STE and pPR3-N; +, positive control, i.e., pBT3-STE and pOstl-NubI; Pns11/actin, pBT3-STE-Pns11 and pPR3-N-actin. **(D)** GST pull-down assay was used to detect interaction between Pns11 of RGDV and actin of *R. dorsalis*. Pns11 of RGDV was fused to His as a bait protein and GFP as a control. Actin of *R. dorsalis* was fused with GST as a prey protein. The GST-fused actin specifically bound to His-fused Pns11, but it did not bind to His-fused GFP. All immunofluorescence figures are representative of at least three repetitions.

The association of Pns11 tubules with actin-based junctions or microvilli suggested that RGDV Pns11 may specifically interact with actin of insect vectors. We previously observed through electron microscopy that Pns11 tubules can move along actin-based cellular protrusions between cultured cells derived from *R. dorsalis*, facilitating viral spread among its insect vector cells (Chen et al., 2013). Immunofluorescence microscopy further revealed that Pns11 tubules were closely associated with the actin filaments in the cytoplasm or on the cellular protrusions of the cultured cells (**Figures 5A,B**). Thus, Pns11 tubules were directly associated with vector actin *in vivo* or *in vitro.* We then used a yeast two-hybrid assay and detected that RGDV Pns11 can directly interact with actin of *R. dorsalis* (**Figure 5C**). In addition, we generated GST-fused actin and His-fused Pns11, and found that GST-actin can pull down His-Pns11, but not His-GFP (**Figure 5D**). These results suggested that the association of Pns11 tubules with the actin-based junctions or microvilli of the vector ovarian cells was mediated by a specific interaction between Pns11 and actin.

### Silencing of Pns11 by RNAi Arrested the Invasion of Oocyte by RGDV

**FIGURE 6:**
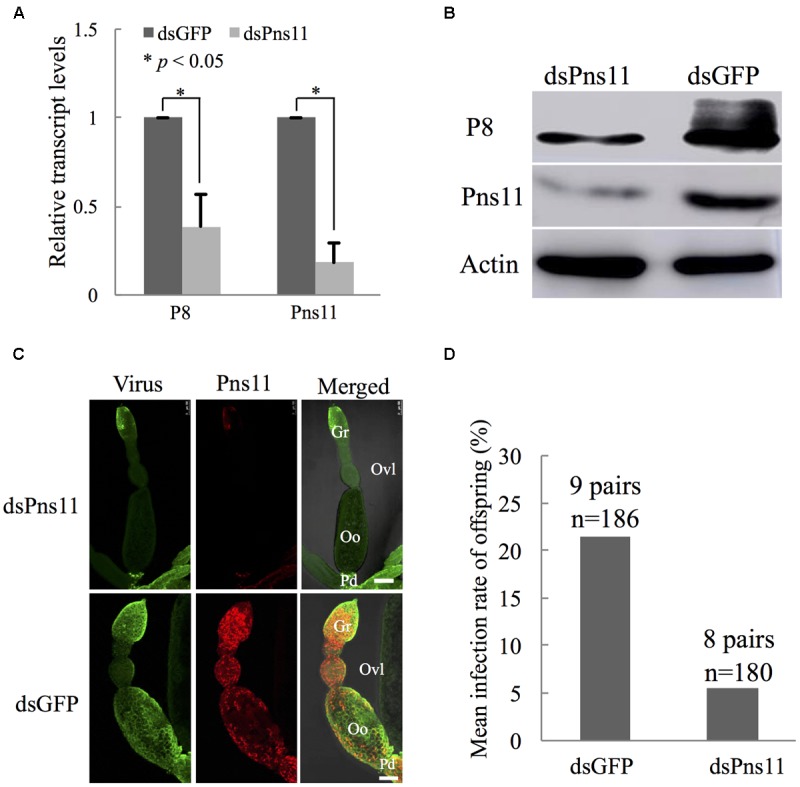
**Microinjection of dsPns11 suppressed the transovarial transmission of RGDV to insect offspring. (A)** At 4 days after dsRNAs microinjection, the effects of dsPns11 or dsGFP on the transcript levels of P8 or Pns11 genes of RGDV in insect ovaries, as revealed by RT-qPCR assay, with mean ± standard deviation (SD) of three independent experiments with standard error, ^∗^*P* < 0.05. **(B)** At 4 days after dsRNAs microinjection, the effects of dsPns11 or dsGFP on the accumulation of P8 or Pns11 of RGDV in insect ovaries, as revealed by Western blot with Pns11- or P8-specific IgGs. Insect actin was detected with actin-specific IgG as a control. **(C)** Effects of dsPns11 or dsGFP treatment on the accumulation of Pns11 tubules and viral infection in insect ovaries, as revealed by immunofluorescence microscopy. At 4 days after dsRNAs microinjection, ovaries were immunolabeled with virus-FITC (green) and Pns11-rhodamine (red), and then processed for immunofluorescence microscopy. Gr, germarium; Oo, oocyte; Ovl, ovariole; Pd, pedicel. Bars: 100 μm. **(D)** Mean RGDV infection rate of offspring from the crosses from dsPns11- or dsGFP-treated viruliferous females with non-viruliferous males. All immunofluorescence figures are representative of at least three repetitions.

To further demonstrate the critical role of Pns11 tubules in viral invasion into the oocyte, we microinjected newly emerged individual viruliferous females with dsPns11 or dsGFP, then excised the ovaries at 4 days after microinjection to extract total RNAs and proteins for relative RT-qPCR and Western blot assays. At 4 days after microinjection, the transcript levels of Pns11 gene or viral major outer capsid protein P8 gene in dsPns11-treated viruliferous ovaries were reduced by about 82 or 61%, respectively (**Figure 6A**). Accordingly, Western blot assay indicated that the accumulation of Pns11 and P8 of RGDV in dsPns11-treated viruliferous ovaries was also significantly decreased (**Figure 6B**). Immunofluorescence microscopy further revealed that the assembly of Pns11 tubules and viral infection in dsPns11-treated viruliferous ovaries were also significantly inhibited (**Figure 6C**). To determine whether the inhibition of Pns11 tubule assembly affected transovarial transmission of RGDV to insect offspring, we crossed dsPns11- or dsGFP-treated virgin females with healthy males and measured viral presence by RT-PCR assay in the F1 offspring. About 5% of the offspring from dsPns11-treated females were viruliferous, compared with 21% from the dsGFP-treated females (**Figure 6D**), showing that the treatment with dsPns11 inhibited viral dissemination in the ovary and reduced viral transmission to the offspring. Taken together, these data suggested that Pns11 tubules played an important role in viral invasion into the oocyte and thus transovarial transmission by insect vectors.

## Discussion

Efficient movement of viral particles into the vector oocyte is critical for transovarially transmitted viruses, yet little is known about the mechanisms by which the virus enters the oocyte. In insect ovaries, the germarium maintains oocyte production and contains germ cells with nuclei of irregular outline (Swiatek, 2006; Szklarzewicz et al., 2007). These germ cells are the initial entry locations for symbiotic bacteria such as *Wolbachia* in *Drosophila melanogaster* and for viral pathogens such as RSV in *Laodelphax striatellus* (Huo et al., 2014; Newton et al., 2015). Here, we characterized the infection route of RGDV to enter the oocyte for transovarial transmission by *R*. *dorsalis* vector. RGDV initially entered the germ cells in the germarium, moved between follicular cells, then translocated across the microvilli to access the oocyte cytoplasm (**Figure 1**). RGDV, *Wolbachia* and RSV seem to share a common infection route from the germarium to the oocyte in their respective vectors.

At 9 days after insect eclosion, we calculated that RGDV had spread into about 80% of intestines, but only into about 21% of ovaries (**Figure 1** and **Table 1**). At this time, viruses had abundantly accumulated in the germarium and follicular cells of virus-infected ovaries. However, RGDV had successfully entered the mature oocytes from follicular cells in only about one-third of virus-infected ovaries (**Figure 1** and **Table 1**). We thus determined that RGDV encountered strong membrane or tissues barriers in its spread paths from the hemocoel to the ovary germarium, then to the follicular cells and finally into the oocytes.

Previously, we have shown that tubules induced by plant reoviruses, such as southern rice black-streaked dwarf virus (SRBSDV) and RDV, can exploit ABTM to spread across the midgut tissues of their insect vectors (Chen et al., 2012; Jia et al., 2014; Wei and Li, 2016). In this study, we found that RGDV can exploit Pns11 tubules carrying viral particles to traverse the actin-based junctions between follicular cells or directly pass through the actin-based microvilli from follicular cells to oocyte cytoplasm of insect vectors (**Figures 2, 3**). We previously used an RNAi strategy to specifically knock down the expression of Pns11 by treatment with dsPns11; the tubules failed to assemble, and viral spread was prevented among cultured insect vector cells without significant effects on viral multiplication (Chen et al., 2013). Here, we further determined that the failure of Pns11 tubule assembly after treatment with dsPns11 significantly inhibited viral entry into the oocyte and thus transovarial transmission by insect vectors (**Figure 6**). Because RGDV exploited Pns11 tubules to spread from initially infected regions in virus-infected ovaries, it was reasonable that the treatment dsPns11 would significantly reduce viral accumulation. Based on these results, we deduced that virus-induced tubule acted as a vehicle for spread of RGDV into ovary oocyte cells. Furthermore, we found Pns11 can specifically interact with actin, the main component of ovary junctions or microvilli, *in vivo* or *in vitro* (**Figure 5**). We thus predicted that Pns11 tubules may be propelled by ABTM to overcome the tissue barriers of actin-based cellular junctions or microvilli of the ovary, finally facilitating viruses to overcome transovarial transmission. Thus, ABTM may be an efficient and common mechanism for plant reoviruses to overcome membrane or tissue barriers within insect vectors.

Whether other insect factors are involved in the ABTM-mediated transovarial transmission of RGDV in its insect vector is still unknown. Recently, we report that Pns10 of RDV, the functional counterpart of Pns11 of RGDV, can specifically interact with actin, tropomoduli, myosin, Vg, and lipophorin precursor of insect vectors (Chen et al., 2015, 2017). Vg is a major yolk protein precursor in insects for the developing oocytes to meet the nutrient requirements during egg development (Zhang et al., 2016). Generally, this female-specific protein also crosses the junctions between follicular cells and is taken up into the oocyte through the microvilli (Tufail and Takeda, 2008, 2009). Thus, we predict that Vg may be involved in the ABTM-mediated transport of Pns11 tubules of RGDV between follicular cells or into the oocyte cytoplasm. Previous data has shown that an endogenous retrovirus of *D. melanogaster*, ZAM, is transmitted from follicular cells to the oocyte by making use of the endogenous endocytic yolk uptake mechanism (Brasset et al., 2006). Similarly, the outer capsid protein of RSV can interact with Vg and use Vg as a vehicle to enter the vector oocyte (Huo et al., 2014). It will be interesting to investigate whether Vg-mediated viral entry into the oocyte is a common mechanism of transovarial transmission.

Previously, we have shown that the infection of RGDV directly affects the viability and development of its vector offspring (Chen et al., 2016; Lan et al., 2016), suggesting that RGDV is harmful to the early insect embryo. Our current study showed that the infection of RGDV induced viroplasm formation due to non-structural protein Pns9 in the oocyte cytoplasm (**Figure 4**). Viroplasm of plant reovirus is the site for viral replication and assembly of progeny virions in insect vectors or plant hosts (Wei and Li, 2016). We thus determined that propagation of RGDV in the early insect embryo may directly cause cytopathologic changes or damage functionally relevant tissues, decreasing the fitness of insect offspring, similar to the cytopathological changes that the plant reoviruses caused in the insect midgut and salivary glands (Wei and Li, 2016). Alternatively, the replication of RGDV can induce small interfering RNA antiviral immunity responses in the oocyte cytoplasm, which may also be compensated by the fitness of insect offspring (Lan et al., 2016).

Many arboviruses such as Dengue and Zika viruses can be vertically transmitted by insect vectors such as mosquitoes (Lequime and Lambrechts, 2014; Grunnill and Boots, 2016; Thangamani et al., 2016), but the mechanism is unclear. Because almost all arboviruses can replicate and induce viral inclusions in respective insect vectors, our current model that the utilization of virus-induced inclusions for viral spread through insect oocyte cytoplasm by RGDV would be extensively exploited by arboviruses, opening new perspectives for viral control.

## Author Contributions

TW: Conceived and designed the experiments. ZL, QM, JL, CL, and WW: Performed the experiments. HC: Maintained insect cell line. QC, DJ, ZL, and QM: Analyzed the data. TW, ZL, and QM: Wrote the manuscript. All authors read and approved the final manuscript.

## Conflict of Interest Statement

The authors declare that the research was conducted in the absence of any commercial or financial relationships that could be construed as a potential conflict of interest.
