# Supplementary material for: Virus-Induced Tubules: A Vehicle for Spread of Virions into Ovary Oocyte Cells of an Insect Vector
Source: Front Microbiol. 2017 Mar 22;8:475. doi: 10.3389/fmicb.2017.00475 (PMC5360704; doi:10.3389/fmicb.2017.00475)
Supplement: Supplementary file 1 [file Table_1.DOCX]

Supplemental table 1. Primers used in this study

| Genes | Primers | Sequences (5*′*-3*′*) |
| --- | --- | --- |
| RGDV P8 | P8-forward | ATGGACGTAGCTAGATCGTCA |
|  | P8-reverse | ACACACTAACTACTAGCAATAGA |
|  | Q-P8-forward | GATTCAAGGGGCACAGAACG |
|  | Q-P8-reverse | GTAATGGTTGCGACTGGGTC |
|  |  |  |
| RGDV Pns11 | Q-Pns11-forward | CCTACCATGGATGCGGACAC |
|  | Q-Pns11-reverse | CCGTCAGCTGTAAGACGCAA |
|  |  |  |
| *R. dorsalis* actin | Q-actin-forward | GCCGTCTTTCTTGGGTATGG |
|  | Q-actin- reverse | GCCGTCTTTCTTGGGTATGG |
